# Physics-Constrained Neural ODEs for MXene Bandgap Prediction with Conformal Uncertainty

**DOI:** 10.3390/nano16110673

**Published:** 2026-05-27

**Authors:** Nida Kati, Ferhat Ucar

**Affiliations:** 1 Metallurgical and Materials Engineering Department, Faculty of Technology, Fırat University, Elazığ 23200, Turkey; 2 Software Engineering Department, Faculty of Technology, Fırat University, Elazığ 23200, Turkey

**Keywords:** neural ODE, physics-constrained learning, Δ learning, conformal prediction, MXene, bandgap, photocatalysis

## Abstract

Two-dimensional transition metal carbides and nitrides, known collectively as MXenes, are attractive photocatalyst candidates because their surface chemistry and atomic composition can be tuned over a wide compositional window. A crucial design quantity is the electronic bandgap, which selects whether a given MXene couples with solar radiation and aligns with the redox levels of water splitting. High-fidelity bandgap calculations using the PBE0 hybrid functional are computationally expensive, which has motivated several machine learning surrogates. To the best of our knowledge, this is the first study to integrate a continuous-depth Neural Ordinary Differential Equation backbone with multi-fidelity Δ learning, distribution-free split-conformal calibration, and uncertainty-aware Pareto screening into a single mathematically grounded pipeline for MXene bandgap prediction. In this work, we develop a physics-constrained neural ordinary differential equation (PC-NODE) that predicts MXene bandgaps from a compact 34-dimensional descriptor set, without relying on the density of states. The model couples a classifier head for the metal/semiconductor decision with a regression head for the gap magnitude, and enforces three physically motivated properties: non-negativity of the predicted gap and monotonicity between the low-fidelity Perdew–Burke–Ernzerhof (PBE) and the high-fidelity PBE0 estimates are obtained exactly through a softplus-parameterised Δ learning construction, while a hurdle coupling that drives metal predictions towards zero is enforced via a quadratic penalty and verified empirically. In short, two of the three physical constraints are guaranteed by construction, and the third is approximately enforced and verified empirically; the same distinction is maintained consistently in the methodology, the constraint audit and the conclusion. Trained on the 4356-structure MXgap database, a ten-seed ensemble reaches a mean absolute error of 0.186 eV (per-seed 0.206±0.006 eV) and a coefficient of determination $R^{2}=0.880$ on the semiconductor test subset, with a classifier accuracy of 0.856 and a Receiver Operating Characteristic Area Under the Curve (ROC-AUC) of 0.925. A split-conformal calibration step then delivers prediction intervals whose empirical coverage matches the 90% target within 0.5 percentage points. Finally, an uncertainty-aware Pareto screening step applies the trained surrogate to a held-out subset of 396 lanthanum-based MXenes and identifies 74 candidates inside the photocatalytic water splitting window [1.23, 3.10] eV. The framework offers a mathematically grounded, data-efficient alternative to feature-heavy pipelines and is reproducible from the open MXgap resource.

## 1. Introduction

Since their discovery through the exfoliation of the MAX phase Ti_3_AlC_2_ [1], MXenes, the family of two-dimensional (2D) transition metal carbides, nitrides and carbonitrides with the general formula $M_{n+1}X_{n}T_{2}$, have emerged as a versatile platform for energy conversion [2,3]. The terminal group *T*, ranging from halogens to chalcogens to hydroxyl and imide moieties, governs surface chemistry and in turn the electronic structure, while the transition metal *M* and the non-metal *X* fix the inner framework. In photocatalytic water splitting and solar CO_2_ conversion, these tunable properties translate into a practical design requirement; the electronic bandgap $E_{g}$ must lie in the window [1.23,3.10] eV, where the lower bound coincides with the redox potential of water splitting and the upper bound marks the edge of the near-ultraviolet region [4,5,6]. Identifying compositions that meet this criterion over the combinatorial MXene space is therefore a central problem for next-generation energy materials.

The bandgap is in principle accessible through density functional theory (DFT), but accuracy depends strongly on the exchange–correlation functional. Semilocal functionals such as Perdew–Burke–Ernzerhof (PBE) systematically underestimate $E_{g}$, while hybrid functionals such as PBE0 correct this bias at a substantially higher computational cost [7]. For a class as large as MXenes, screening thousands of compositions at the PBE0 level is therefore impractical and has motivated the development of machine learning (ML) surrogates. Early work by Rajan et al. [8] demonstrated that kernel and Gaussian process models could predict functionalised MXene bandgaps from simple elemental descriptors. More recently, data-driven studies have leveraged neural network surrogates for crystal property prediction [9] and applied them to MXenes in particular [10].

The most directly relevant benchmark in this space is the MXgap framework of Ontiveros et al. [11], which released an openly available dataset of 4356 MXene structures and a classifier–regressor pipeline that reports a reported classification accuracy of 92% and a mean absolute error (MAE) of 0.17 eV for $E_{g}$ prediction. Their feature set blends periodic table indices, elemental descriptors and the PBE-level density of states (DOS), totalling more than one hundred variables. Around this benchmark, several adjacent studies have appeared: Tang et al. [10] report MAE of 0.14 eV using a deep high-throughput screening pipeline; Zhang [12] benchmarks gradient boosting, LightGBM and support vector regression on MXene bandgaps, and identifies the binary flag “IsGap” as the most important predictor, which in fact leaks the classification label into the regression inputs; Dolz et al. [13] extend similar ideas to single-metal-atom adsorption energies; and the broader landscape is surveyed by Hjiri and Mustapha [14]. A common thread across these studies is a high-dimensional feature set and, in most cases, a lack of mathematical guarantees on physical consistency or predictive coverage.

From a mathematical standpoint, three ingredients are increasingly seen as enabling rigor in data-driven materials modeling. First, physics-informed neural networks embed constraints derived from physical laws directly into the learning objective [15]. Second, continuous-depth architectures based on neural ordinary differential equations (Neural ODEs) [16] and their augmented variants [17] offer a principled alternative to discrete deep stacks, with adaptive depth and smooth representations. Third, split-conformal inference provides distribution-free coverage certificates for regression predictions, independent of model family [18]. Complementing these ideas, multi-fidelity learning schemes such as Δ learning correct cheap approximations with learned residuals [19], while Bayesian optimisation [20,21] and deep ensembles [22,23] support uncertainty-aware search over candidate materials. These tools are now routinely brought to bear on heterogeneous applied problems, from deep learning for semiconductor band structure reconstruction [24] and physics-informed multi-objective optimisation in power electronics [25] to frequency learning hybrids for power system forecasting [26], artificial intelligence pipelines for soil compaction control [27] and explainable machine learning methods for manufacturing defect prediction [28].

Each of these tools, however, comes with characteristic strengths and limitations that motivate the specific way in which we combine them in this work. *Physics-informed neural networks* make physical laws part of the loss but enforce the constraints only *softly*; there is no *a priori* guarantee that a trained network respects them at test time. *Neural ODEs* replace discrete depth by a continuous flow, which yields adaptive resolution and a smooth latent representation, but their training requires repeated calls to a numerical solver and can be sensitive to step size choices. *Split-conformal inference* delivers a finite-sample, distribution-free coverage guarantee that is agnostic to the underlying model, but it relies on exchangeability between calibration and test residuals and only certifies marginal (rather than conditional) coverage. Δ *learning* exploits a cheap low-fidelity baseline and is highly data-efficient when the baseline is informative, but it transfers a portion of the bias of the baseline to the surrogate. Finally, *Bayesian optimisation* and *deep ensembles* provide principled uncertainty estimates for candidate ranking but at the cost of either a kernel design choice (Bayesian optimisation with Gaussian processes) or a multiplicative training budget (deep ensembles). The PC-NODE framework introduced below is designed precisely so that these strengths reinforce each other while their limitations are mitigated by architecture or by a complementary tool—non-negativity and PBE/PBE0 monotonicity become *architectural* guarantees rather than soft penalties; the Δ learning baseline is paired with the conformal layer that calibrates the residual; and the deep-ensemble layer is reduced to a fixed budget of ten seeds rather than a sliding hyperparameter.

To the best of our knowledge, this is the first study to integrate a continuous-depth Neural ODE backbone with multi-fidelity Δ learning, distribution-free split-conformal calibration, and uncertainty-aware Pareto screening into a single mathematically grounded pipeline for MXene bandgap prediction. Building on these threads, we propose a framework that brings a mathematically grounded pipeline to the MXene bandgap problem. We combine a continuous-depth Neural ODE backbone with a softplus-parameterised Δ learning bridge between PBE and PBE0 that delivers two physical properties exactly, a penalised hurdle coupling that drives metal predictions towards zero, split-conformal intervals for distribution-free coverage, and an uncertainty-aware Pareto screening step that operates on a held-out family of lanthanum-based MXenes. Throughout, we use a compact 34-dimensional descriptor set that excludes the DOS, so that predictions can be made from readily available elemental and geometric information alone.

Our main contributions are threefold:

- We introduce a physics-constrained neural ODE (PC-NODE) architecture with a classifier–regressor coupling whose softplus-parameterised Δ learning head guarantees, by construction, two physical properties of the bandgap prediction: non-negativity ${\hat{E}}_{g}^{\mathrm{PBE}0}\geq 0$ and monotonicity ${\hat{E}}_{g}^{\mathrm{PBE}0}\geq E_{g}^{\mathrm{PBE}}$ with respect to the low-fidelity estimate. A third, hurdle, condition that metal predictions collapse toward zero is imposed through a quadratic penalty and verified empirically.
- We pair this surrogate with split-conformal prediction to obtain distribution-free coverage certificates, and verify that the empirical coverage of the reported intervals matches their nominal level on a held-out test split.
- We apply the trained model to a held-out set of 396 lanthanum-based MXenes that was never seen during training, validation or calibration, and run an uncertainty-aware Pareto screening step that combines the predicted bandgap, the classifier probability and the Monte Carlo dropout spread to rank 74 candidates predicted to lie inside the photocatalytic window [1.23, 3.10] eV.

## 2. Materials and Methods

### 2.1. Dataset

We use the openly available MXgap dataset [11], which collects 4356 MXene structures of the generic stoichiometry $M_{n+1}X_{n}T_{2}$. The transition metal *M* is drawn from {Sc, Y, Ti, Zr, Hf, V, Nb, Ta, Cr, Mo, W}, the non-metal *X* from {C, N}, and the terminal group *T* from {F, Cl, Br, I, O, S, Se, Te, H, OH, NH}. The index *n* ranges over 1–3 and six relative stacking geometries are considered per composition, yielding a full factorial design with eighteen structures per (M,X,T) combination and 4356 entries in total. The dataset additionally contains a held-out set of 396 lanthanum-based MXenes, which we reserve exclusively for out-of-distribution screening in Section 4.

Each record carries two electronic targets: the binary label IsGap∈{0,1}, which distinguishes metals from semiconductors, and the high-fidelity hybrid-functional bandgap $E_{g}^{\mathrm{PBE}0}$ in electronvolts. The low-fidelity PBE estimate $E_{g}^{\mathrm{PBE}}$ is also provided and serves as an input in our multi-fidelity scheme (Section 2.4). Across the full set, 3410 records are classified as metals and 946 as semiconductors, producing a class imbalance of approximately 78:22 that motivates a weighted classification loss. The bandgap distribution among semiconductors is strongly right-skewed, with mean 0.96 eV and median 0.67 eV; 226 of the 946 semiconducting structures (24%) fall inside the photocatalytic window [1.23,3.10] eV. We also note, in anticipation of the Δ learning analysis of Section 2.4, that only two of the 946 semiconducting records exhibit a marginally negative $\delta =E_{g}^{\mathrm{PBE}0}-E_{g}^{\mathrm{PBE}}$ (minimum −0.076 eV), so the PBE ≤ PBE0 inequality holds on 99.8% of the dataset; the softplus parameterisation that we use later enforces this inequality exactly on predictions.

**Figure 1:**
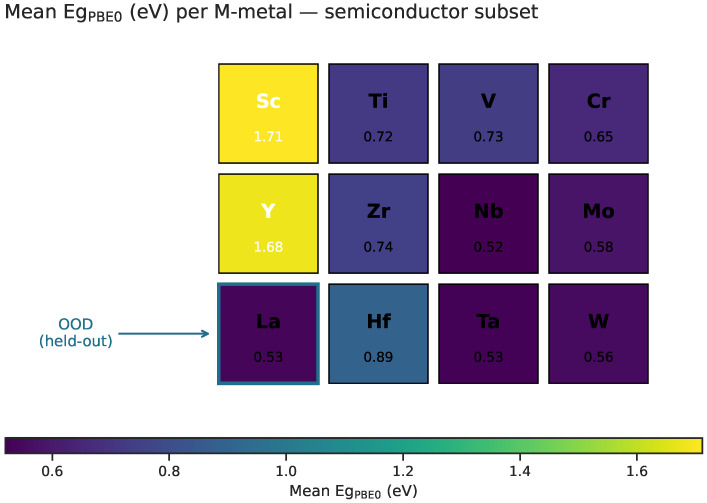
Mean $E_{g}^{\mathrm{PBE}0}$ per transition metal *M* in the MXgap dataset, restricted to the semiconductor subset. Group III metals (Sc and Y) produce the largest mean bandgaps. Lanthanum (La) is present only in the held-out subset, indicated by the *Out-of-Distribution* (OOD) annotation, and is used in Section 4.9 for screening.

**Figure 2:**
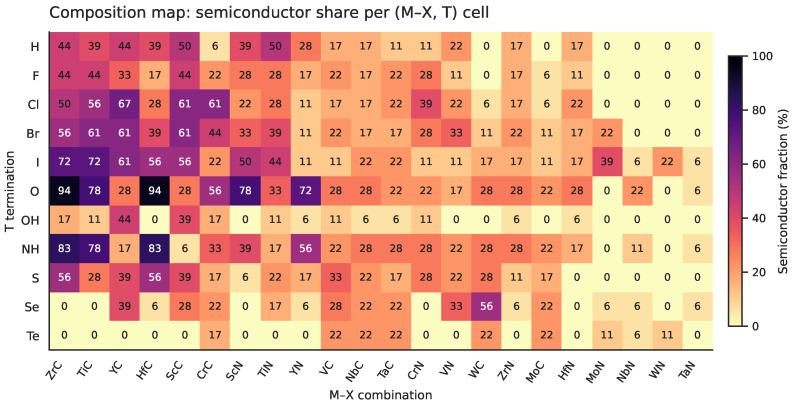
Composition map showing the fraction of semiconductors per (M–X,T) cell. Each cell value is the percentage of structures in the full factorial design (18 per cell) that carry a nonzero PBE0 bandgap. Darker cells identify chemistries that almost always yield metals.

Figure 1 places each transition metal in its periodic table position and colours it by the mean $E_{g}^{\mathrm{PBE}0}$ of its semiconducting subset. Group III metals (Sc and Y) generate the largest mean bandgap, while group V and VI metals tend to produce narrow-gap semiconductors; lanthanum, which appears only in the held-out subset, is annotated separately. Figure 2 then projects the semiconductor–metal split onto the (M–X,T) plane, where each cell reports the fraction of semiconducting structures for the corresponding chemistry. The heterogeneous pattern confirms that bandgap existence is a chemistry-dependent phenomenon rather than a uniform effect and justifies the joint classifier–regressor treatment used throughout this work.

**Figure 3:**
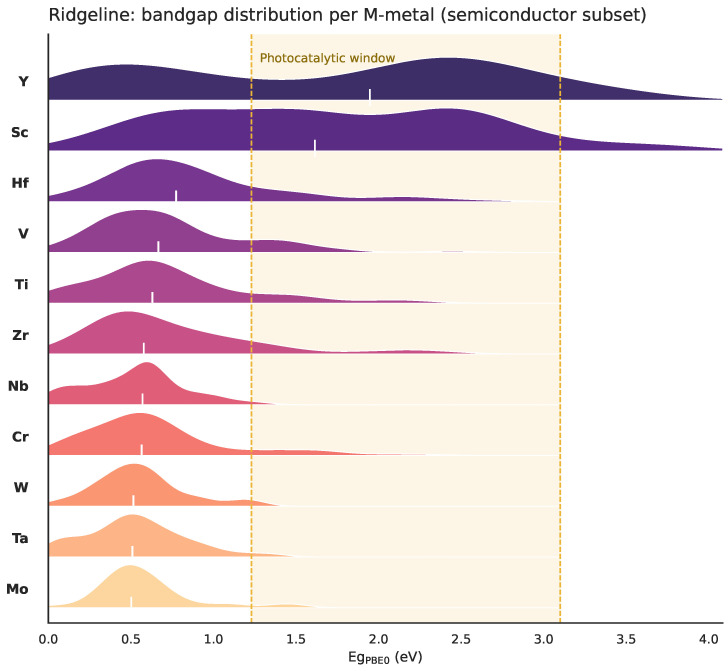
Ridgeline distributions of $E_{g}^{\mathrm{PBE}0}$ for the semiconductor subset, stratified by transition metal *M* and ordered by median. The shaded band marks the photocatalytic water splitting window [1.23,3.10] eV; vertical ticks denote per-metal medians.

**Figure 4:**
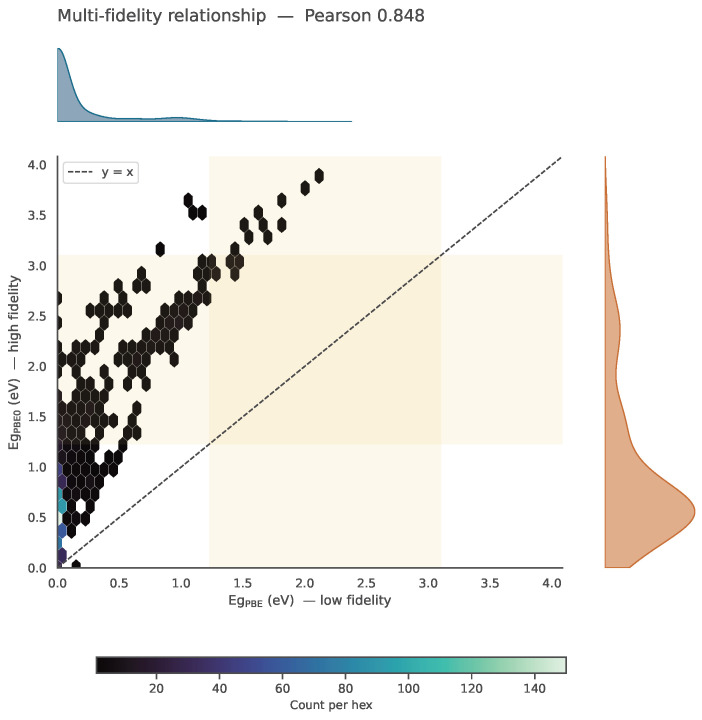
Hexagonal density of the relationship between the PBE and PBE0 bandgaps on the semiconductor subset. The dashed diagonal marks the identity line; the shaded orthogonal bands indicate the photocatalytic window. Marginal kernel density estimates are shown at the top and right.

The distribution of semiconducting bandgaps per transition metal is shown as a ridgeline plot in Figure 3. Ordering the metals by median $E_{g}^{\mathrm{PBE}0}$ highlights a clear structure: Sc and Y dominate the upper end of the range and deposit most of their mass inside the photocatalytic window, whereas W, Ta, Mo and the rest cluster near low values. Finally, Figure 4 visualises the relationship between the low-fidelity $E_{g}^{\mathrm{PBE}}$ and the high-fidelity $E_{g}^{\mathrm{PBE}0}$ on the semiconductor subset. The Pearson correlation is 0.848 and the PBE values lie systematically below the PBE0 values, confirming the well-known underestimation of semilocal functionals and motivating the multi-fidelity Δ learning formulation introduced in Section 2.4.

Two practical details are worth noting. First, the $E_{g}^{\mathrm{PBE}}$ column of the lanthanum sheet in the raw spreadsheet is corrupted by a string-concatenation artefact in the original export. Because the PBE bandgap is algebraically identical to the difference between the conduction band minimum and the valence-band maximum, we reconstruct it from the clean ${\mathrm{CBM}}_{\mathrm{PBE}}$ and ${\mathrm{VBM}}_{\mathrm{PBE}}$ columns. On the training sheet, this reconstruction is accurate to numerical precision (residual ≤ $10^{-16}$ eV), giving us confidence that the same rule applies to the held-out subset. Second, we use the dataset without additional filtering to preserve reproducibility from the original resource.

### 2.2. Descriptor Set

The MXgap resource provides four naturally distinct feature blocks: periodic table indices (atomic number, period, and group for *M*, *X* and *T*, nine variables in total), elemental descriptors (electronegativity, electron affinity, van der Waals radius and atomic radius for each of the three positions, twelve variables), geometric descriptors (layer count *n*, stack and hollow indices, in-plane lattice constant *a*, out-of-plane spacing *d*, and several *M*–*T* and *X*–*T* bond distances, thirteen variables), and a 100-dimensional PBE-level DOS vector. Combined, they amount to 134 variables.

Throughout this work we rely on a *compact* descriptor set that concatenates the periodic table, elemental and geometric blocks and excludes the DOS, giving p=34 input variables.

This choice is motivated by four complementary considerations.

*(i) Physical interpretability of the retained blocks.* Each of the three retained blocks has a distinct physical meaning. The periodic table block (nine variables) encodes the periodic table position of *M*, *X* and *T* and provides the most coarse-grained chemical fingerprint of an MXene. The elemental block (12 variables) refines this picture with continuous atomic properties (electronegativity, electron affinity, van der Waals and atomic radii) for each of the three positions. The geometric block (13 variables) captures the structural identity of the MXene through the layer count *n*, the stacking and hollow indices, the in-plane lattice constant *a*, the out-of-plane spacing *d*, and the *M*–*T* and *X*–*T* bond lengths that determine local bonding. Together, these three blocks are the descriptor classes that can be assembled without first running a self-consistent DFT calculation on the candidate structure.

*(ii) Computational cost.* The DOS block is, by construction, an output of a DFT calculation; using it as input for a surrogate that is meant to reduce DFT cost would partly defeat its purpose. The periodic table, elemental and geometric blocks are, by contrast, available from the chemical formula and a single relaxed geometry, which is inexpensive relative to a full electronic structure run.

*(iii) Information overlap with the multi-fidelity baseline.* The Δ learning parameterisation of Equation (2) already uses the PBE-level bandgap $E_{g}^{\mathrm{PBE}}$ as a scalar baseline; this is one of the same quantities that the DOS block contributes through its bandgap edge channels. Part of the information that a DOS feature vector would carry is therefore already exploited by the architecture, but at the cost of a single scalar rather than a 100-dimensional vector.

*(iv) Empirical evidence on this dataset.* The empirical comparison reported in the PC-NODE ensemble study of Section 4.2 confirms that, on the MXgap data and at the regularisation budget used here, augmenting the compact set with twelve DOS principal components increases the test MAE from 0.186 eV to 0.217 eV, and the full DOS configuration moves further to 0.233 eV. Adding DOS information here therefore degrades rather than improves generalisation, in line with the feature-to-sample-ratio analysis below.

The resulting feature-to-sample ratio is about 1:90 for the full 4356 structure pool and about 1:20 on the 946-record semiconductor subset that drives the regression target; both ratios are favourable for the small training partition used in this study. A complementary, model-driven validation of the compact set is provided by the permutation feature importance analysis reported in Section 4.5; it shows that the geometric and elemental blocks together carry the bulk of the predictive signal, while the periodic table indices play a secondary role.

**Figure 5:**
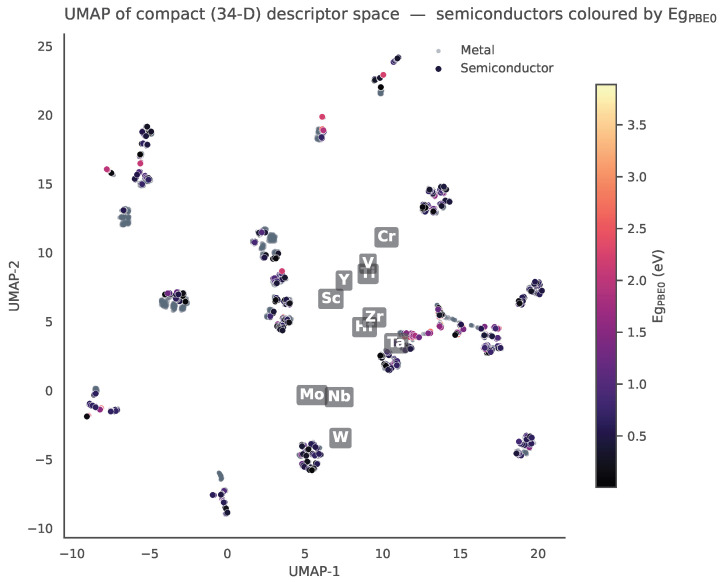
UMAP projection of the compact 34-dimensional descriptor space. Metallic entries are shown as grey circles; semiconducting entries are colour-coded by $E_{g}^{\mathrm{PBE}0}$. Dominant per-metal clusters are labelled.

Figure 5 projects the compact descriptors into two dimensions using a Uniform Manifold Approximation and Projection (UMAP) embedding. Because UMAP does not preserve global geometry in a metric sense, we interpret the resulting layout as a suggestive visual motivation rather than a proof of cluster separability; still, the organisation of points by transition metal, and the structured position of semiconducting members within each neighbourhood, is consistent with the view that chemistry already explains a large share of the variance in $E_{g}^{\mathrm{PBE}0}$.

### 2.3. Physics-Constrained Neural ODE

Let $x\in R^{p}$ denote the compact descriptor vector for a given MXene structure and $y_{\mathrm{low}}=\max\left(0,E_{g}^{\mathrm{PBE}}\right)$ the clamped low-fidelity bandgap. We seek a map that predicts both the classification target $\hat{p}\left(y_{\mathrm{cls}}=1|x\right)$ and the high-fidelity bandgap ${\hat{E}}_{g}^{\mathrm{PBE}0}$, and that respects three physically motivated properties. Two of them, non-negativity and PBE ≤ PBE0 monotonicity, are obtained exactly by the architectural parameterisation described below; a third, the hurdle condition that metal predictions should collapse towards zero, is imposed through a quadratic penalty and therefore holds only approximately at optimum. Embedding physical constraints inside Neural ODE backbones is a recent and active research direction, with closely related Physics-Informed Neural ODE formulations recently reported for chemical engineering kinetics [29] and physics-informed ML reviewed at materials science scale by [30].

**Figure 6:**
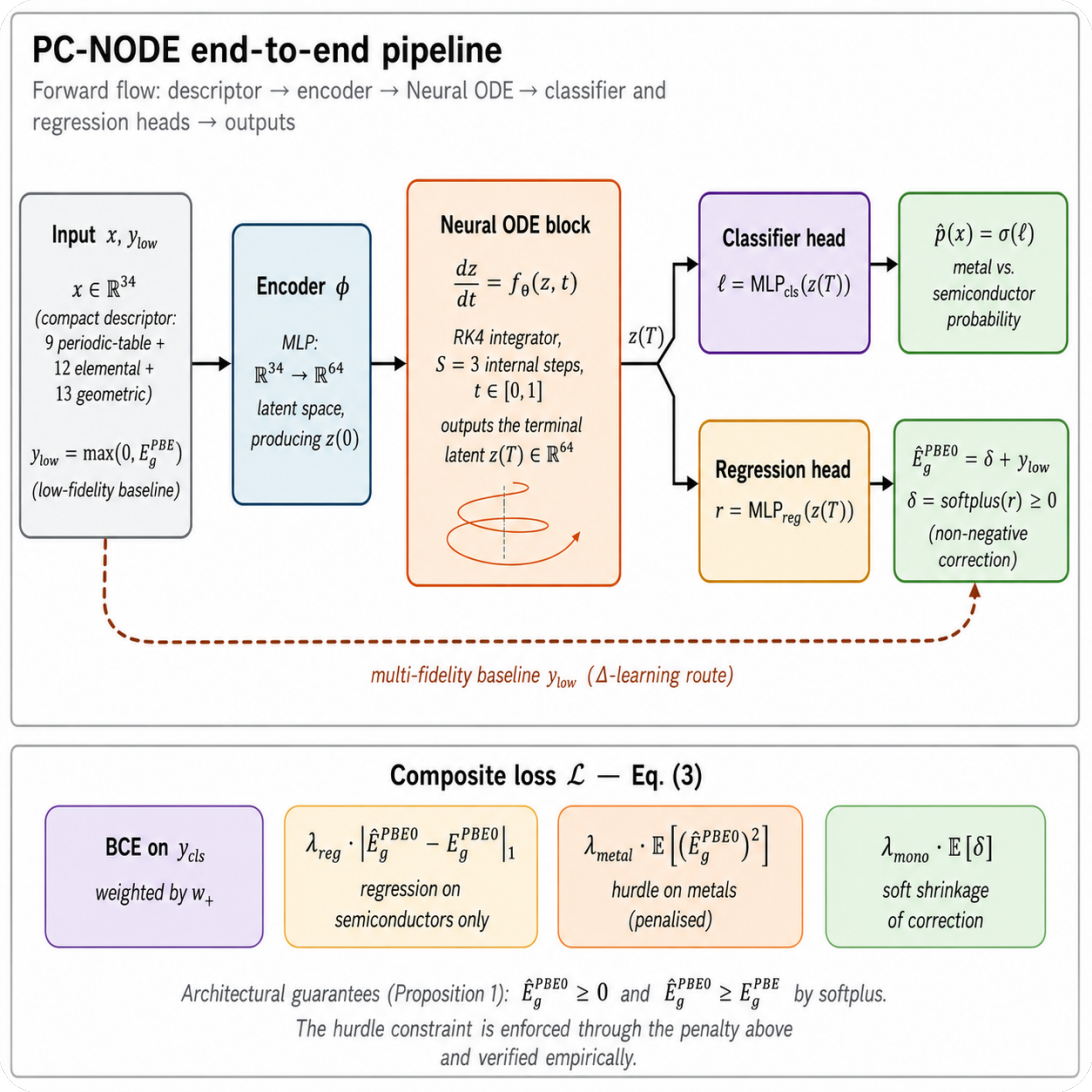
Architecture schematic of the physics-constrained neural ODE. The compact descriptor x is mapped through an encoder φ to a latent state, evolved by the Neural ODE block of Equation (1), and read out at z(T). A classifier head returns the metal/semiconductor probability $\hat{p}$, while a regression head produces a softplus-transformed correction δ that is added to the clamped low-fidelity baseline $y_{\mathrm{low}}=\max\left(0,E_{g}^{\mathrm{PBE}}\right)$ to yield the high-fidelity prediction ${\hat{E}}_{g}^{\mathrm{PBE}0}$. The combined parameterisation enforces non-negativity and PBE ≤ PBE0 monotonicity by construction (Proposition 1); the hurdle condition that drives metal predictions toward zero is added as a penalty in the loss of Equation (3).

The full architecture is summarised in Figure 6. Two inputs enter the pipeline: the compact descriptor x and the scalar low-fidelity baseline $y_{\mathrm{low}}$. The descriptor is mapped through an encoder φ to a latent state $z\left(0\right)\in R^{d}$, evolved over a unit time interval by the Neural ODE block governed by the velocity field $f_{\theta }$, and read out at z(T). Two linear heads share this terminal latent: the classifier head outputs the metal/semiconductor probability $\hat{p}$ through a logistic sigmoid, while the regression head produces a raw scalar *r* that is passed through a softplus to give the non-negative Δ learning correction δ. The high-fidelity bandgap prediction is then obtained by adding δ to the clamped baseline $y_{\mathrm{low}}$. Because $y_{\mathrm{low}}\geq 0$ and δ>0, the resulting prediction is automatically non-negative and at least as large as the PBE estimate, the two architectural guarantees of Proposition 1. The remainder of this subsection makes each block of the schematic precise.

Following the Neural ODE formalism of Chen et al. [16,17], the model first encodes the descriptor through an affine–nonlinear encoder $\phi :R^{p}\to R^{d}$ and then evolves the resulting latent state $z\left(t\right)\in R^{d}$ along a continuous-time trajectory defined by a velocity field $f_{\theta }$. As it is stated in Equation (1):

(1) $$\frac{dz(t)}{dt}=f_{\theta }\;\left(z(t),t\right),\;z\left(0\right)=\phi \left(x\right),$$

where $f_{\theta }$ is parameterised by a small multi-layer perceptron with parameters θ and the terminal state z(T) carries the learned representation. We integrate over a fixed unit interval with a fourth-order Runge–Kutta scheme; the number of internal steps *S* is a hyperparameter that controls the effective depth of the representation.

The terminal state is passed through two linear heads. The classifier head produces a logit ℓ(z(T)), and the regression head outputs a raw scalar r(z(T)) that is transformed into a non-negative correction δ. Equation (2) shows how the predicted bandgap is assembled by summing the clamped low-fidelity estimate with the softplus-transformed correction:

(2) $${\hat{E}}_{g}^{\mathrm{PBE}0}\left(x\right)=\mathrm{softplus}\;\left(r(z(T))\right)+y_{\mathrm{low}},\;\hat{p}\left(x\right)=\sigma \;\left(\ell (z(T))\right),$$

where σ is the logistic sigmoid and $\mathrm{softplus}\left(u\right)=\log\left(1+e^{u}\right)>0$ for all u∈R. Writing δ(x)=softplus(r(z(T))), we have δ(x)>0 and $y_{\mathrm{low}}\geq 0$, so the architecture yields ${\hat{E}}_{g}^{\mathrm{PBE}0}\left(x\right)>y_{\mathrm{low}}\geq 0$ for every input. This establishes Proposition 1 below, which makes the two architectural guarantees explicit.

**Proposition** **1** (Architectural guarantees)**.** *Under the parameterisation of Equation (2), with $y_{\mathrm{low}}=\max\left(0,E_{g}^{\mathrm{PBE}}\right)$, the following properties hold for every input x and every $E_{g}^{\mathrm{PBE}}\in R$:*

(i) *Non-negativity. ${\hat{E}}_{g}^{\mathrm{PBE}0}\left(x\right)>0$.*
(ii) *PBE ≤ PBE0 monotonicity. ${\hat{E}}_{g}^{\mathrm{PBE}0}\left(x\right)>E_{g}^{\mathrm{PBE}}$.*

**Proof.**  By definition $\mathrm{softplus}\left(u\right)=\log\left(1+e^{u}\right)>0$ for every u∈R; therefore δ(x)>0. Claim (i) follows because $y_{\mathrm{low}}\geq 0$ and ${\hat{E}}_{g}^{\mathrm{PBE}0}=\delta +y_{\mathrm{low}}>0$. For Claim (ii), if $E_{g}^{\mathrm{PBE}}\geq 0$ then $y_{\mathrm{low}}=E_{g}^{\mathrm{PBE}}$ and ${\hat{E}}_{g}^{\mathrm{PBE}0}=y_{\mathrm{low}}+\delta >y_{\mathrm{low}}=E_{g}^{\mathrm{PBE}}$; if $E_{g}^{\mathrm{PBE}}<0$, the clamp gives $y_{\mathrm{low}}=0$ and ${\hat{E}}_{g}^{\mathrm{PBE}0}=\delta >0>E_{g}^{\mathrm{PBE}}$. □

**Remark** **1** (empirical activation of the clamp)**.** *Although the proof of Proposition 1 accommodates the $E_{g}^{\mathrm{PBE}}<0$ case for completeness, in the MXgap database every reported PBE bandgap is non-negative; across all 4356 training records (both 3410 metals and 946 semiconductors) and all 396 records of the lanthanum-based held-out subset of Section 4.9, $E_{g}^{\mathrm{PBE}}\geq 0$ with no exceptions. Consequently the clamp $y_{\mathrm{low}}=\max\left(0,E_{g}^{\mathrm{PBE}}\right)$ reduces to the identity $y_{\mathrm{low}}=E_{g}^{\mathrm{PBE}}$ on every observed input, and introduces* no bias to the semiconductor predictions *of this study. The clamp is retained in the parameterisation as a defensive mechanism; it preserves the architectural guarantees of Proposition 1 on hypothetical out-of-distribution inputs whose PBE bandgap might be returned as a small negative value by some DFT implementations as a numerical band overlap artefact. A separate, distinct point concerns the inequality $E_{g}^{\mathrm{PBE}}\leq E_{g}^{\mathrm{PBE}0}$, which is violated for two of the 946 semiconducting records (with $\delta =E_{g}^{\mathrm{PBE}0}-E_{g}^{\mathrm{PBE}}\geq -0.08$ eV); these cases are clamped by the strictly positive softplus on δ rather than by the $\max(0,E_{g}^{\mathrm{PBE}})$ operation, and we discuss them further in Section 2.4.*

The hurdle condition ${\hat{E}}_{g}^{\mathrm{PBE}0}\approx 0$ for metals is not an architectural guarantee, because softplus is strictly positive and $y_{\mathrm{low}}$ can be strictly positive for metallic inputs; it is instead enforced *penally* by the loss term introduced below, and its empirical efficacy is reported in Section 4.6.

The training objective combines a weighted binary cross-entropy (BCE) term on the classification label with a regression term that is applied only on the semiconductor subset, a hurdle penalty that pushes the predicted bandgap towards zero for metals, and a small regulariser that favours compact corrections. The composite loss is given in Equation (3):

(3) $$\begin{array}{cc} L\;=\; & \mathrm{BCE}\;\left(\ell ,y_{\mathrm{cls}};\;w_{+}\right)\\ \; & \;+\;\lambda_{\mathrm{reg}}\;E_{y_{\mathrm{cls}}=1}\;\left|{\hat{E}}_{g}^{\mathrm{PBE}0}-E_{g}^{\mathrm{PBE}0}\right|\\ \; & \;+\;\lambda_{\mathrm{metal}}\;E_{y_{\mathrm{cls}}=0}\;{\left({\hat{E}}_{g}^{\mathrm{PBE}0}\right)}^{2}\\ \; & \;+\;\lambda_{\mathrm{mono}}\;E\;\delta , \end{array}$$

where $w_{+}$ is the positive-class weight that compensates for the 78:22 imbalance, the $\lambda_{\mathrm{reg}}$ term is an $\ell_{1}$ loss on the regression output restricted to semiconductors, the $\lambda_{\mathrm{metal}}$ term is the hurdle penalty that drives metal predictions toward zero, and the $\lambda_{\mathrm{mono}}$ term is a weak shrinkage on the correction δ that prefers small Δ learning updates whenever they are not required by the data. The multipliers $\lambda_{\mathrm{reg}}$, $\lambda_{\mathrm{metal}}$ and $\lambda_{\mathrm{mono}}$ are small positive constants whose values are listed in Section 3.

**Figure 7:**
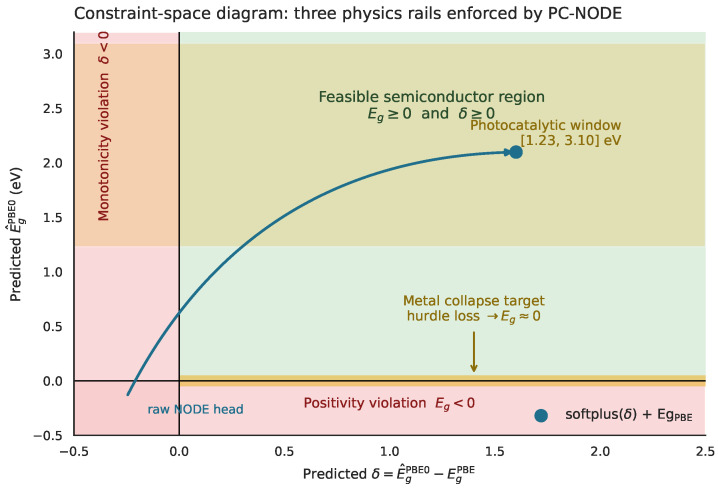
Constraint space of the PC-NODE. The feasible region (green) corresponds to non-negative ${\hat{E}}_{g}^{\mathrm{PBE}0}$ and non-negative δ; violations would sit in the pink regions but are excluded by the softplus parameterisation. The yellow band near the horizontal axis marks the hurdle region where metal predictions are driven. The arrow illustrates the transformation of the raw Neural ODE output into the feasible region.

Figure 7 summarises the resulting constraint space. The predicted pair $\left(\delta ,{\hat{E}}_{g}^{\mathrm{PBE}0}\right)$ always lies in the upper-right quadrant as a consequence of Proposition 1; the hurdle term pulls metal predictions toward the horizontal axis, and only predictions that additionally fall inside the photocatalytic band are of practical interest for water splitting.

### 2.4. Multi-Fidelity Δ Learning

The parameterisation of Equation (2) is a neural instantiation of the Δ learning idea of Ramakrishnan et al. [19], an approach that has continued to gain traction in quantum chemical property prediction; for example, Ghosh et al. [31] have recently used Δ machine learning to correct triplet excitation energy predictions of organic chromophores against TDDFT references; the cheaper low-fidelity signal $E_{g}^{\mathrm{PBE}}$ is used as a baseline and the network is asked to learn only the non-negative residual δ that reconciles PBE with the more expensive PBE0 value. This construction carries three practical benefits. First, the learning problem is easier because the baseline already captures most of the variance (the Pearson correlation between PBE and PBE0 on the semiconductor subset is 0.848, as shown in Figure 4). Second, the constraint δ≥0 is consistent with the general tendency of hybrid functionals to produce bandgaps that are at least as large as those of their semilocal counterparts; in our training set this inequality is violated in only two out of 946 semiconducting records, both with δ≥−0.08 eV, and these narrow cases are forced to δ=0 in prediction by the softplus clamp, introducing a small but bounded bias. Third, the same parameterisation applies verbatim to the lanthanum-based held-out subset, where $E_{g}^{\mathrm{PBE}}$ is available but $E_{g}^{\mathrm{PBE}0}$ is not.

### 2.5. Split-Conformal Prediction

A point prediction of the bandgap is rarely sufficient in materials design, where downstream decisions involve costly synthesis or characterisation. We supplement the PC-NODE surrogate with split-conformal intervals [18] that deliver finite-sample marginal coverage regardless of the underlying estimator. Conformal prediction has been increasingly adopted in scientific machine learning for the same reason: a recent example in molecular ML is the interpretable conformal-and-counterfactual framework of Ghamsary et al. [32] for selective inhibitor design. Concretely, we partition the training data into a model-fitting subset and a calibration subset; after training on the former, we evaluate absolute residuals $r_{i}=\left|E_{g,i}^{\mathrm{PBE}0}-{\hat{E}}_{g,i}^{\mathrm{PBE}0}\right|$ on the latter and obtain the empirical quantile as shown in Equation (4):

(4) $${\hat{q}}_{\alpha }=r_{\left(\left\lceil \left(n_{\mathrm{cal}}+1\right)\left(1-\alpha \right)\right\rceil \right)},$$

where $n_{\mathrm{cal}}$ denotes the number of calibration residuals and $r_{\left(k\right)}$ is the *k*-th order statistic. The prediction interval at a new input $x_{★}$ then follows from Equation (5):

(5) $${\hat{C}}_{\alpha }\left(x_{★}\right)=\left[\max\left(0,{\hat{E}}_{g}^{\mathrm{PBE}0}\left(x_{★}\right)-{\hat{q}}_{\alpha }\right),\;{\hat{E}}_{g}^{\mathrm{PBE}0}\left(x_{★}\right)+{\hat{q}}_{\alpha }\right],$$

where the lower bound is clipped at zero to respect the physical non-negativity of bandgaps. Under exchangeability of calibration and test residuals, the resulting interval covers the true $E_{g}^{\mathrm{PBE}0}$ with probability at least 1−α. We emphasise explicitly that this coverage guarantee is conditional on exchangeability between the calibration and test residuals: it transfers to a held-out test subset that is drawn from the same distribution as the calibration set, but it does *not* automatically transfer to the lanthanum-based out-of-distribution subset of Section 4.9, where the chemistry of the inputs lies outside the training pool. The consequences of this distinction are made operational in Section 4.9, where a formally correct treatment would require weighted or nonexchangeable conformal variants [33], and revisited in the limitations paragraph of Section 5.

### 2.6. Uncertainty-Aware Pareto Screening

Given a trained PC-NODE surrogate, we frame the screening of photocatalytic candidates as a two-objective optimisation problem. We describe this step as *uncertainty-aware Pareto screening* rather than Bayesian optimisation because no sequential acquisition loop is involved: the surrogate is trained once and is then queried on a candidate library, and the two objectives are ranked jointly through Pareto dominance and a scalar tie-breaker. For a candidate MXene structure x with PBE estimate $E_{g}^{\mathrm{PBE}}\left(x\right)$, the window distance is defined in Equation (6):

(6) $$d_{\mathrm{window}}\left(x\right)=\max\;\left(E_{\mathrm{lo}}-{\hat{E}}_{g}^{\mathrm{PBE}0}\left(x\right),\;{\hat{E}}_{g}^{\mathrm{PBE}0}\left(x\right)-E_{\mathrm{hi}},\;0\right),$$

where $E_{\mathrm{lo}}=1.23$ eV and $E_{\mathrm{hi}}=3.10$ eV delimit the photocatalytic band. The second objective is the negative classifier probability $-\hat{p}\left(x\right)$, so that minimising both simultaneously favours candidates whose predicted bandgap falls inside the water splitting window *and* for whom the model is confident that they are semiconducting. The Pareto front over the candidate library is obtained by standard non-dominated sorting. A secondary ranking inside the front is provided by the augmented Chebyshev scalarisation [21], given in Equation (7):

(7) $$s_{\tau }\left(x\right)=\max\;\left(\tau_{1}\;d_{\mathrm{window}}\left(x\right),\;-\tau_{2}\;\hat{p}\left(x\right)\right)+\rho \;\left(\tau_{1}\;d_{\mathrm{window}}\left(x\right)-\tau_{2}\;\hat{p}\left(x\right)\right),$$

where $\tau_{1},\tau_{2}>0$ are weights, ρ>0 is a small augmentation term and low values of $s_{\tau }$ indicate preferred candidates. Epistemic uncertainty on the two objectives is propagated from the PC-NODE surrogate through Monte Carlo (MC) dropout [23], following common practice for uncertainty estimation in deep models [22]. We stress that MC dropout captures only epistemic, model-level uncertainty; it is known to underestimate predictive uncertainty in out-of-distribution regimes [34,35], and we therefore use it in this work as a qualitative tie-breaker rather than as a calibrated error bar. We make the two methodological caveats associated with this choice explicit. First, MC dropout estimates *epistemic* uncertainty—the variability of the network output under random masks of its hidden units—and does *not* attempt to capture aleatoric uncertainty arising from intrinsic noise in the data. Second, in out-of-distribution regimes such as the lanthanum-based subset of Section 4.9, the variance of the dropout ensemble is known to be a poor proxy for the true predictive uncertainty and tends to *underestimate* it [34,35]. The split-conformal intervals of Section 2.5 therefore remain the primary, distribution-free quantitative coverage tool of the framework, and the MC dropout spread is reported throughout the paper only as a qualitative diagnostic. The framework therefore combines three uncertainty tools, each selected for a specific role: a ten-seed deep ensemble (in the sense of Lakshminarayanan et al. [22]; see the PC-NODE ensemble study of Section 4.2) reduces seed-induced variance in the point predictions; the split-conformal step delivers calibrated, distribution-free intervals; and MC dropout produces a cheap per-candidate epistemic spread for use as a tie-breaker on the secondary screening objective. We adopt MC dropout rather than an additional independent-ensemble layer or a Gaussian-process surrogate for two reasons. First, the headline PC-NODE numbers already exploit a deep ensemble of ten independently trained PC-NODE models (Section 4.2; per-seed standard deviation 0.006 eV) and an additional ensemble layer dedicated to the per-candidate spread would be largely redundant given that scale. Second, an exact Gaussian process surrogate scales as $O(n^{3})$ in the training set size and would, on the 4356-structure MXgap pool, require either a kernel design that the present continuous-depth backbone deliberately avoids or sparse approximations whose own coverage behaviour would have to be re-examined; the MC dropout pass, in contrast, integrates seamlessly with the dropout layers already present in the PC-NODE architecture and adds no additional training cost. Quantitative coverage guarantees on ${\hat{E}}_{g}^{\mathrm{PBE}0}$ come from the split-conformal intervals of Section 2.5; the MC dropout spread is reported alongside them as a diagnostic signal. We apply the pipeline to the 396-structure lanthanum-based held-out subset in Section 4.9.

## 3. Experimental Setup

Before turning to empirical results, we describe the computational environment, the train/validation/test protocol and the hyperparameters used to obtain all numbers reported in this study. Every run is reproducible from the open MXgap resource [11] and the accompanying scripts.

### 3.1. Data Splits

All models are trained on the 4356-structure training pool described in Section 2.1. For the main benchmark we use a stratified 70/15/15 partition into training, validation and test subsets, with stratification by the binary label $y_{\mathrm{cls}}$ to preserve the metal/semiconductor ratio across splits. The 396-structure lanthanum-based subset remains untouched in all stages of model development and is only examined in the out-of-distribution screening of Section 4.9. For the split-conformal calibration in Section 2.5, the training pool is further divided in half into a model-fitting subset and a calibration subset, following the standard split-conformal recipe of Lei et al. [18].

### 3.2. Model Hyperparameters

The PC-NODE architecture introduced in Section 2.3 is instantiated with a single configuration for all reported numbers. Table 1 lists the model and optimiser hyperparameters; they were selected via a small grid on the validation subset and kept fixed thereafter. The ensemble is produced by re-running the same configuration over ten random seeds (11, 23, 47, 89, 137, 211, 313, 401, 503 and 601), with predictions averaged at test time.

### 3.3. Software and Hardware

All experiments were run in a Miniconda virtual environment on a single workstation equipped with an Apple M3 Max processor (16 cores) and 64 GB of unified memory, under macOS 26.4. Neural network computations were executed on the Metal Performance Shaders (MPS) backend of PyTorch. The software stack used Python 3.12, PyTorch 2.11.0, torchdiffeq 0.2.5 for the Neural ODE integration, scikit-learn 1.7.2 for baselines and data splitting [36], pandas 2.3.3 and numpy 2.3.4 for data handling, UMAP-learn for the low-dimensional embedding reported in Section 2.2, and matplotlib and seaborn for figures. All random seeds were fixed at script level; the ten seeds used for the ensemble are listed in Section 3.2.

## 4. Results

We first report classical baselines across the four descriptor sets of Section 2.2, then describe the tuned PC-NODE ensemble, audit the physics constraints, report the split-conformal coverage, and finally apply the inverse design pipeline first to the full library and then to the lanthanum-based held-out subset.

### 4.1. Baseline Comparison Across Descriptor Sets

**Figure 8:**
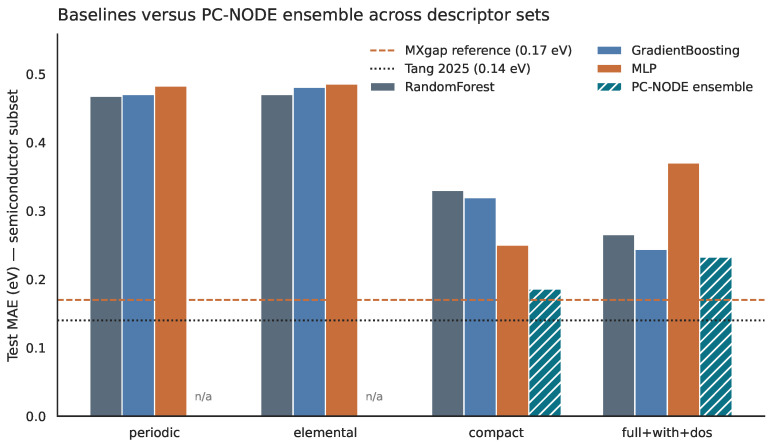
Test MAE of the three baselines (RF, GBT, MLP) across the four descriptor sets, together with the PC-NODE ensemble on the compact and full + DOS sets. Horizontal dashed and dotted lines mark the reference values of MXgap [11] and Tang et al. [10] respectively.

As a reference for later comparisons, we train Random Forest (RF), Gradient Boosting (GBT) and Multi-Layer Perceptron (MLP) regressors on each of the four descriptor sets defined in Section 2.2. To enable an apples-to-apples comparison with the PC-NODE ensemble, all baselines use the same stratified 70/15/15 split introduced in Section 3.1—each model is trained on the PC-NODE training partition, and evaluated on the PC-NODE test partition. The regression task is restricted to the semiconductor subset of each partition, following the MXgap recipe. Figure 8 summarises the test MAE of each (model,descriptor) combination and juxtaposes them against two reference values from the literature: the 0.17 eV MAE reported by the MXgap framework [11] and the 0.14 eV MAE reported by the deep learning screening pipeline of Tang et al. [10].

Two observations stand out. First, the periodic and elemental-only sets are too shallow for any of the baselines to reach a competitive MAE, with all three models settling between 0.46 and 0.49 eV. Second, the compact set already brings a marked improvement, the MLP reaching 0.250 eV on this shared test split and the tree-based models hovering near 0.32 eV. The full + DOS set brings a further small improvement for the tree-based baselines (GBT reaches 0.244 eV and RF 0.265 eV) but it also makes the MLP noticeably worse (0.370 eV), which we attribute to the much larger feature-to-sample ratio in that regime. Already at this stage, the best baseline MAE on the shared test split is 0.244 eV, which leaves room for the PC-NODE ensemble reported in the next subsection to close further toward the MXgap reference.

### 4.2. Tuned PC-NODE Ensemble

With the baseline picture in place, Table 2 reports the PC-NODE ensemble results over three descriptor settings. Each row aggregates ten training runs with different random seeds, averaged at test time; we report both the per-seed mean and the ensemble-level metric. On the compact set, the ensemble attains a test MAE of 0.186 eV and $R^{2}=0.880$, which closes most of the gap to the MXgap reference of 0.17 eV while using only 34 input features and no DOS information. Extending the input by twelve DOS principal components actually degrades the ensemble MAE to 0.217 eV, and the full + DOS configuration does worse still (0.233 eV). This is a reversal of the typical pattern: for PC-NODE, adding DOS features degrades generalisation on this dataset. The reversal likely stems from two complementary factors. First, the regularisation budget used for the compact set may be insufficient for a 134-dimensional input when only a few thousand training samples are available. Second, the DOS vector is an auxiliary output of the same PBE calculation that produces $E_{g}^{\mathrm{PBE}}$, so at least part of the target information is already encoded in the low-fidelity baseline used by our Δ learning parameterisation, which reduces the marginal value of the DOS block. We therefore use the compact configuration for all subsequent analyses. A cross-descriptor ensemble that averages the three settings gives an intermediate MAE of 0.200 eV, confirming that the compact set dominates the averaging.

**Figure 9:**
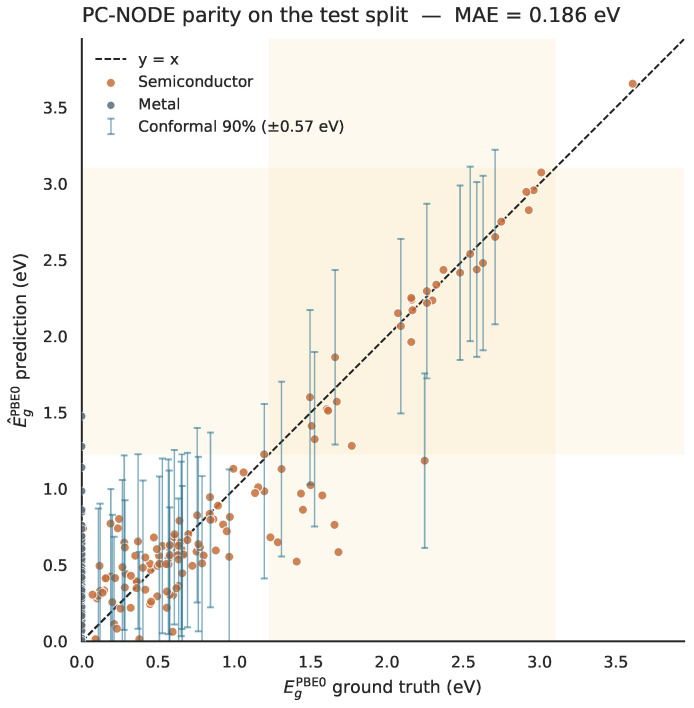
Parity between PC-NODE ensemble predictions and PBE0 ground truth on the test split. Metallic structures (dark grey markers), whose PBE0 reference is zero by definition, cluster along the vertical axis at ground truth =0; their predicted gaps are pulled toward zero by the hurdle term and therefore lie close to the origin. Semiconductors (orange markers) scatter along the diagonal. Blue error bars on a random subset of semiconductor points denote 90% conformal intervals.

Figure 9 visualises the parity between predicted and true PBE0 bandgaps for the compact ensemble on the held-out test split. Metallic structures, whose PBE0 reference value is zero by construction, cluster along the vertical axis at $E_{g}^{\mathrm{PBE}0}=0$, with the predicted gaps pulled toward zero by the hurdle term in Equation (3); they appear as the dark cluster of points hugging the *y*-axis in the lower left of the figure. Semiconductors scatter along the diagonal with residuals that remain compact across the whole range [0,4] eV. Conformal error bars (see Section 4.7) are overlaid on a random subset of forty semiconductor points to give a visual sense of the predictive uncertainty.

To quantify the residual structure that is visible on the parity plot we compute the per-bin MAE, bias and residual standard deviation on the 142 semiconductors of the test split: [0.0,0.5) eV, N=44, MAE 0.202 eV, bias +0.133 eV, residual std 0.220 eV; [0.5,1.0) eV, N=51, MAE 0.129 eV, bias −0.092 eV, residual std 0.149 eV; [1.0,2.0) eV, N=24, MAE 0.353 eV, bias −0.321 eV, residual std 0.336 eV; [2.0,3.0) eV, N=21, MAE 0.111 eV, bias −0.072 eV, residual std 0.235 eV; [3.0,4.0) eV, N=2, MAE 0.054 eV, bias +0.054 eV, residual std 0.009 eV; pooled (all bins) MAE 0.186 eV, bias −0.020 eV, residual std 0.271 eV.

Three observations emerge that nuance the visual reading of Figure 9. First, the largest per-bin MAE (0.353 eV) and the strongest systematic bias (−0.321 eV) lie in the [1,2) eV bin, that is, at the lower edge of the photocatalytic window of Equation (6), and *not* at small ground-truth values. Two factors contribute: this bin contains only 24 of 142 test semiconductors, so the empirical residual is the noisiest, and the bin sits in the right tail of the training δ distribution of Figure 4, where the Δ learning correction is asked to produce its largest values from a comparatively sparse training signal. We flag this regime as a natural target for future data-augmentation efforts. Second, in the small-gap region ([0,0.5) eV) the residual is positive on average (+0.133 eV bias), reflecting a mild tendency of the surrogate to push narrow-gap semiconductors away from zero by the same hurdle pressure that drives metals towards it. This bias is small relative to the overall MAE and does not affect the metal/semiconductor separation at the classifier level. Third, the visual impression that the conformal error bars grow at small ground-truth values is an artefact of the constant-width construction of Equation (5); the calibrated half-width ${\hat{q}}_{0.10}=0.57$ eV is identical for every point, but the lower bound is clipped at zero to respect the physical non-negativity of bandgaps, which makes the bars *appear* asymmetric near zero even though their calibrated half-width is unchanged. We therefore interpret the figure not as “higher uncertainty at small gaps” but as “constant-width conformal intervals on a heteroscedastic residual landscape whose dominant deviation is concentrated near the photocatalytic-window boundary.”

### 4.3. Classifier Performance

While the regression MAE of Table 2 is the headline metric, the classifier head of PC-NODE matters in two distinct ways: it drives the hurdle penalty that collapses metal predictions toward zero, and it produces the $\hat{p}$ signal that is used as the second objective in the Pareto screening of Section 4.8. Table 3 reports its performance on the shared test split. The ensemble reaches 0.856 accuracy and 0.925 ROC-AUC, with a macro $F_{1}$ of 0.810 and a recall of 0.831 on the minority semiconductor class. The lower precision (0.628) corresponds to 70 false positives out of 654 test structures; the positive-class weight $w_{+}$ used in the binary cross-entropy loss deliberately trades some precision for recall to counteract the 78:22 class imbalance. For reference, the MXgap framework [11] reports a classification accuracy of 0.92; our classifier head gives up some accuracy relative to that reference but does not rely on the DOS features.

### 4.4. Ablation Study

To isolate the contribution of each ingredient of PC-NODE, we train four variants on the same 70/15/15 stratified split over five random seeds and report the resulting per-seed mean and standard deviation of the test MAE on the semiconductor subset. The variants are:

- **A1—Plain MLP (regression only).** A feed-forward network trained directly on the semiconductor subset, without a classifier head, a Δ learning parameterisation or a hurdle penalty. This is the closest conceptual analogue of classical baselines that regress PBE0 in a single shot.
- **A2—MLP + softplus hurdle.** Same feed-forward backbone, but the regression head is replaced by the softplus Δ learning parameterisation of Equation (2) and trained jointly with the classifier using the full loss of Equation (3).
- **A3—Neural ODE without physics.** The continuous-depth backbone is restored but both the softplus non-negativity constraint and the hurdle penalty are removed; the regression head outputs an unconstrained scalar.
- **A4—Full PC-NODE.** The configuration of Table 2, with both the continuous-depth backbone and the physics constraints.

Table 4 reports the MAE and the constraint diagnostics for each variant. Three observations stand out. First, the single largest MAE reduction comes from replacing the classical regression-only MLP (A1, 0.248 eV) with a joint classifier–regressor whose regression head uses the softplus Δ learning parameterisation of Equation (2) (A2, 0.203 eV). Second, removing the physics constraints from the Neural ODE backbone (A3) degrades the MAE only marginally relative to the full PC-NODE (A4), but it produces 6 to 66 positivity violations per seed and a minimum δ down to −0.59 eV—that is, the unconstrained NODE actively violates the physics of the problem, which is exactly what motivates the architectural design. Third, the difference between the hurdle-based MLP (A2, 0.203±0.010 eV) and the full PC-NODE (A4, 0.214±0.016 eV) lies within the per-seed standard deviations of the two variants, so we do not read it as a statistically significant gap; both configurations satisfy Proposition 1 by construction, whereas A3 does not. Taken together, the ablation identifies the softplus Δ learning parameterisation as the single most consequential architectural choice. The Neural ODE backbone then provides a continuous-depth, smooth representation that preserves these physical guarantees at comparable predictive accuracy. The headline ensemble result of Table 2 (MAE =0.186 eV over ten seeds) sits below both the A2 and A4 per-seed averages because ensemble averaging reduces variance across seeds.

The qualitative meaning of the constraint diagnostics in Table 4 can be summarised by a side-by-side comparison of the predicted PBE0 bandgap distributions of variants A3 (unconstrained) and A4 (full PC-NODE) over the same five seeds, illustrated in the Discussion (Section 5). The unconstrained variant produces a left tail that crosses zero and accumulates 184 predictions in the physically forbidden region ${\hat{E}}_{g}^{\mathrm{PBE}0}<0$ (with a minimum of −0.588 eV), whereas the PC-NODE places no mass in that region; every test prediction satisfies ${\hat{E}}_{g}^{\mathrm{PBE}0}\geq 0$ as a consequence of the softplus parameterisation of Equation (2). The narrow A4 spike at ${\hat{E}}_{g}^{\mathrm{PBE}0}\approx 0$ corresponds to metal samples that are pulled toward zero by the hurdle penalty. The two histograms therefore make the same point that Table 4 reports numerically, but in a form that does not require reading the diagnostics column-by-column.

To complement this architectural ablation with a feature budget-matched comparison against the MXgap reference of Ontiveros et al. [11], we additionally evaluated the same PC-NODE backbone on the larger feature regime that they use—the full descriptor set augmented with the 100-dimensional DOS block, totalling 134 features—on the identical 70/15/15 stratified split used throughout this paper. This provides a direct comparison in which only the feature regime changes between configurations: the model architecture, the train/validation/test split, the optimiser and the seed protocol are held fixed. On this MXgap-equivalent feature regime, the PC-NODE ensemble reaches a test MAE of 0.232 eV with a classification accuracy of 0.798, i.e., *worse* than the compact configuration (0.186 eV MAE, 0.856 accuracy) reported in Table 2, which uses only 34 features. The compact configuration therefore wins the comparison both on accuracy and on simplicity in our fixed-architecture, fixed-split regime; the residual gap to the published MXgap MAE of 0.17 eV reflects differences in their classifier–regressor pipeline (RF/GBT/SV/MLP cascade) and split protocol (60/20/20), as we already discuss in the Discussion (Section 5). We note that a strict identical-checkpoint comparison would require the trained MXgap models in a redistributable form, which is not available from the public Zenodo record; the present feature regime-matched comparison is the closest faithful proxy that we are able to run.

The mechanism behind the deterioration that we observe when DOS information is added to the PC-NODE input is best understood as a combination of two effects, of which the first is dominant. (i) *Information overlap with the* *Δ* *learning baseline.* The PBE-level bandgap is one of the most informative DOS-derived quantities, and our architecture already exploits it: it enters Equation (2) as the Δ learning baseline $y_{\mathrm{low}}$ rather than as an additional feature. Once that scalar is accounted for, the remaining DOS channels add dimensionality faster than they add new predictive signal. (ii) *Curse of dimensionality on neural-network surrogates.* The same direction of change is visible in the plain-MLP baseline of Section 4.1 (Figure 8), whose test MAE deteriorates from 0.250 eV on the compact set to 0.370 eV on the full + DOS set; the tree-based baselines, with their built-in feature selection inductive bias, do not suffer from the same effect. The intermediate DOS-PCA configuration mentioned in the limitations paragraph of Section 5 probes the same regime with a moderate dimensionality penalty and reaches the same qualitative conclusion. We therefore read the deterioration not as overfitting in the strict sense, but as the combination of an architectural redundancy specific to Δ learning surrogates and the standard high-dimensional penalty on neural networks.

### 4.5. Permutation Feature Importance

**Figure 10:**
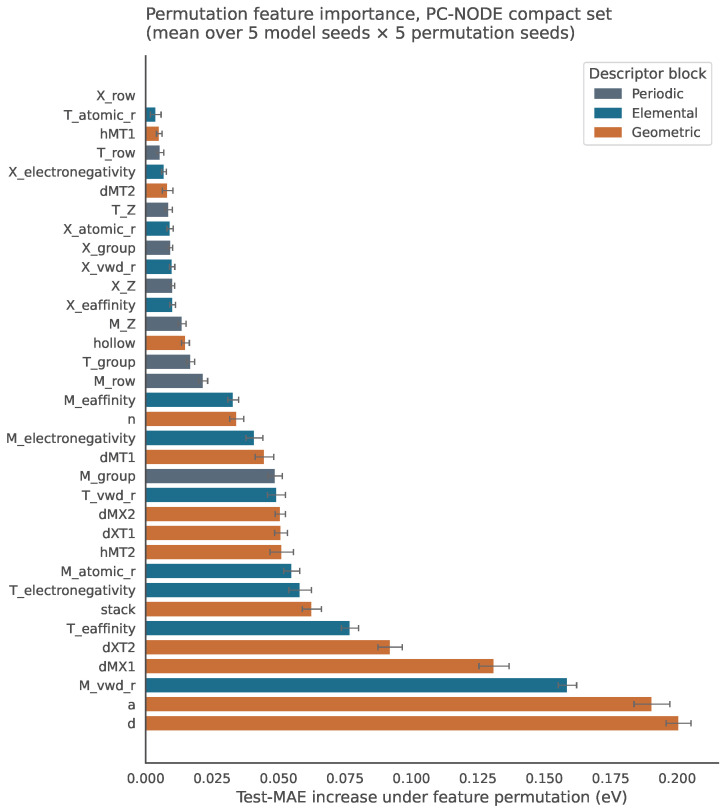
Permutation feature importance for the PC-NODE on the compact descriptor set. Each bar reports the mean increase in test MAE on the semiconductor subset when the corresponding feature is randomly permuted, averaged over five training seeds and five permutation seeds; error bars denote the standard error across seeds. Bars are colour-coded by descriptor block (periodic, elemental, geometric).

To complement the qualitative justification of the compact descriptor set in Section 2.2, we report a permutation-based feature importance analysis on the trained PC-NODE. For each of the p=34 input features we shuffle the corresponding column of the test split, run inference, and measure the resulting increase in test MAE on the semiconductor subset. The procedure is repeated over five independent permutation seeds for each of five training seeds, so that each reported value is the mean of 25 paired comparisons against the per-seed baseline. The result is summarised in Figure 10.

Three observations emerge. First, the model’s most informative inputs are the structural ones: the out-of-plane spacing *d*, the in-plane lattice constant *a* and the *M*–*X* and *X*–*T* bond distances dominate the ranking, with permutation-induced MAE increases of 0.20, 0.19, 0.13 and 0.09 eV respectively. This is consistent with the well-known dependence of the bandgap on local bonding geometry. Second, the elemental properties of the transition metal *M* and the terminal group *T* form a secondary tier, led by the van der Waals radius of *M* (0.16 eV), the electron affinity and electronegativity of *T* (0.08 and 0.06 eV), and the atomic radius of *M* (0.06 eV); these are the continuous descriptors that fine-tune the chemical fingerprint. Third, the periodic table indices for the non-metal *X* contribute very little (permutation increase of ≲0.01 eV), which is expected because *X* takes only two values (C or N) and the same information is already captured at finer resolution by the elemental properties of *X*. Taken together, these observations support the descriptor set choice made in Section 2.2: the geometric and elemental blocks carry the bulk of the predictive signal, the periodic table block contributes redundant but inexpensive context, and the DOS block is not required for the task.

### 4.6. Physics-Constraint Audit

The two architectural guarantees of Proposition 1 are structural properties of the parameterisation and therefore require no empirical check: for every test-set prediction, positivity violations and negative δ values are exactly zero. What remains to be audited empirically is the hurdle condition, which is only a penalised term in the loss of Equation (3) and is not guaranteed by the architecture because softplus(r)>0 and $y_{\mathrm{low}}$ can be strictly positive even for metallic inputs. On the test split, the mean absolute predicted bandgap on metal structures is approximately 0.045 eV, compared with the dataset-wide semiconductor mean of 0.96 eV. This is about twenty times smaller than the regression target scale, which we interpret as evidence that the hurdle penalty is sufficiently strong to collapse metal predictions towards zero in practice, without being so strong as to distort the semiconductor regression.

### 4.7. Conformal Prediction Intervals

**Figure 11:**
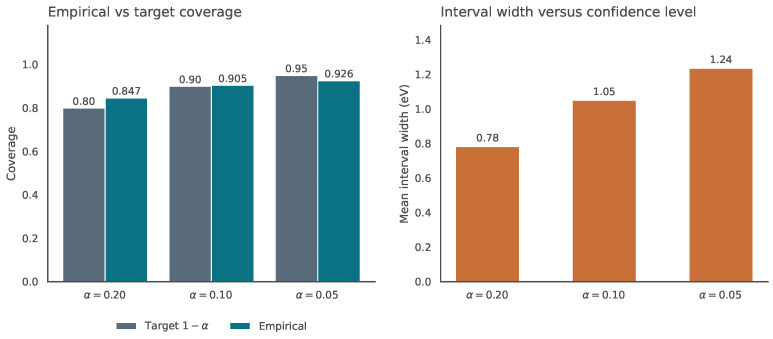
Split-conformal intervals for the PC-NODE regressor. (**Left**): empirical vs. target coverage at the three nominal levels. (**Right**): mean interval width at each level. Both curves behave as expected under exchangeability of calibration and test residuals.

Split-conformal calibration is carried out as described in Section 2.5, with the training subset halved evenly into a model-fitting portion and a calibration portion. Table 5 reports the calibrated quantile ${\hat{q}}_{\alpha }$, the mean interval width and the empirical coverage at the three nominal levels 1−α∈{0.80,0.90,0.95}. Figure 11 visualises both panels. At the 0.90 nominal level, the empirical coverage is 0.905, within 0.5 percentage points of the target, at an interval width of approximately 1.05 eV. The 0.80 level is slightly over-covered at 0.847, and the 0.95 level slightly under-covered at 0.926; both patterns are consistent with a finite calibration set.

### 4.8. Uncertainty-Aware Pareto Screening on the Full Library

**Figure 12:**
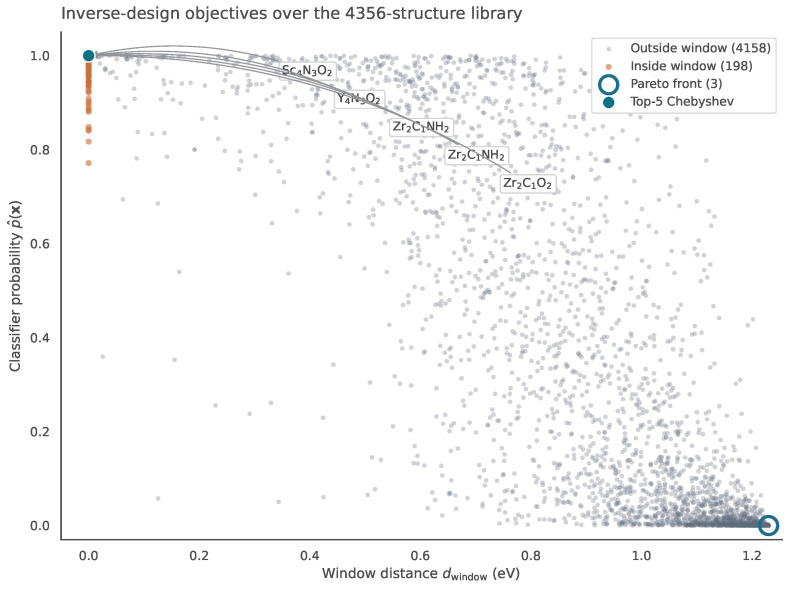
Inverse design objectives over the 4356-structure library. The *x*-axis is the window distance defined in Equation (6); the *y*-axis is the classifier probability $\hat{p}$. Structures inside the photocatalytic window are shown in orange. Blue circles mark Pareto-front members. The top five Chebyshev-ranked candidates are labelled.

Turning the trained surrogate into a design tool, we apply the pipeline of Section 2.6 to the complete 4356-structure training library as a *re-scoring* exercise—every entry becomes a candidate that the surrogate places on the $\left(d_{\mathrm{window}},\;-\hat{p}\right)$ plane. We emphasise that this step is a screening application of a model that has already seen a large fraction of these entries during training, so the resulting candidate counts are not a test of generalisation; an honest cross-validation of the screening output is offered by the held-out lanthanum subset of Section 4.9. Figure 12 presents the joint distribution of the two objectives, highlights the Pareto front and annotates the five top-ranked candidates according to the augmented Chebyshev scalarisation of Equation (7). Of the 4356 structures, 198 are predicted to lie strictly inside the photocatalytic window [1.23,3.10] eV; three of those also lie on the Pareto front. The top Chebyshev-ranked candidate is an Sc_4_N_3_O_2_ composition with a predicted gap of 2.06 eV and classifier confidence $\hat{p}\approx 1$. Because this structure sits inside the training library, its reference PBE0 value is also available: 2.13 eV, so the PC-NODE prediction deviates by 0.07 eV, consistent with the magnitude of typical in-sample residuals produced by the ensemble. The next four candidates are structurally closely related (Y_4_N_3_O_2_, Zr_2_C_1_(NH)_2_ with two geometries, Zr_2_C_1_O_2_), reflecting that both chemistry and stacking geometry modulate the gap inside this family.

### 4.9. Lanthanum-Based Out-of-Distribution Screening

The lanthanum-based subset of 396 structures was not used during training, validation or calibration and offers an informative out-of-distribution (OOD) test; lanthanum sits in Group III alongside Sc and Y (already seen in training) but its 4f–5d electronic character is absent from the training pool. We run the trained PC-NODE surrogate on this subset with Monte Carlo dropout enabled, so that a per-sample standard deviation is produced alongside the point prediction. Of the 396 structures, 132 are classified as semiconductors with $\hat{p}\geq 0.5$ and 74 of those fall inside the photocatalytic window.

Two cautions accompany these numbers. First, MC dropout captures only epistemic, model-level uncertainty; its tendency to underestimate predictive uncertainty in OOD regimes [34,35] means that the reported mean standard deviation of 0.097 eV should be read as an internal-consistency diagnostic rather than as a calibrated error bar. Second, the split-conformal guarantee of Section 4.7 rests on exchangeability between calibration and test residuals and therefore does not automatically transfer to the lanthanum subset; a formally correct treatment of this case calls for weighted or nonexchangeable conformal variants [33], which we flag as a natural extension of the present work.

**Figure 13:**
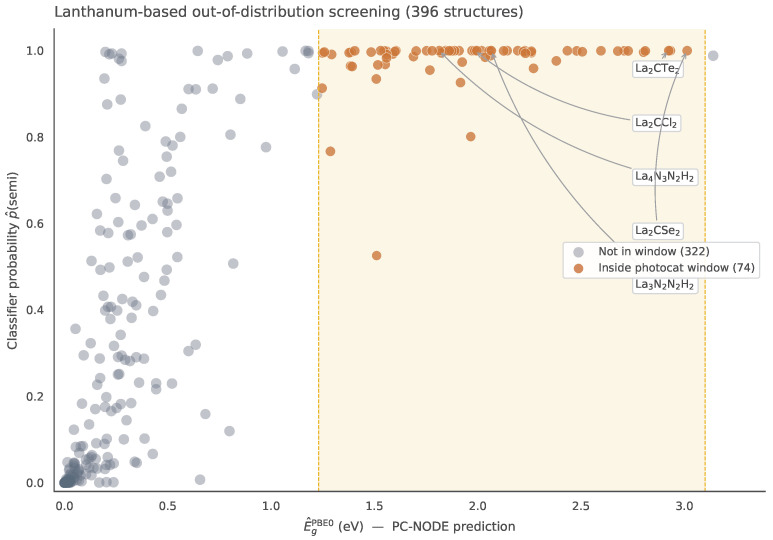
PC-NODE predictions on the 396-structure lanthanum-based subset. Marker size is inversely related to the Monte Carlo dropout standard deviation, so that larger markers correspond to higher model confidence. The shaded vertical band marks the photocatalytic window; the five top candidates listed in Table 6 are labelled.

Table 6 lists the five highest-ranked candidates by classifier confidence; Figure 13 visualises the full subset on the $\left({\hat{E}}_{g}^{\mathrm{PBE}0},\;\hat{p}\right)$ plane. The top candidates combine a tellurium, chlorine or selenium termination with a carbide or nitride backbone, echoing the Sc/Y-rich behaviour observed in the training library. We note that the classifier probabilities saturate at $\hat{p}>0.999$ for all five candidates; this is a consequence of sigmoid saturation rather than an independent signal of confidence, and it is another reason why predictive uncertainty on the OOD subset should be interpreted with care. The La_4_N_3_(NH)_2_ entry in particular combines a zero $E_{g}^{\mathrm{PBE}}$ with a predicted ${\hat{E}}_{g}^{\mathrm{PBE}0}=1.813$ eV, so the required Δ correction sits in the upper tail of the training distribution and the associated MC dropout spread (σ=0.185 eV) is roughly twice that of the other entries; we mark this case explicitly in Table 6 and flag its predicted gap as requiring independent validation.

## 5. Discussion

**Figure 14:**
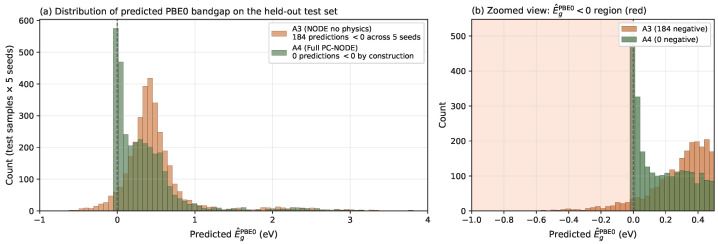
Distribution of predicted PBE0 bandgaps on the held-out test split for the unconstrained Neural ODE (variant A3, orange) and the full PC-NODE (variant A4, green), aggregated across the same five seeds reported in Table 4. Panel (**a**) shows the full distribution over [−1,4] eV; panel (**b**) zooms into the ${\hat{E}}_{g}^{\mathrm{PBE}0}<0$ region (shaded red), which is physically forbidden. The unconstrained variant places 184 predictions in the negative region across the five seeds (minimum $\delta_{\min}=-0.588$ eV), whereas the PC-NODE places exactly 0 predictions there as a consequence of Proposition 1. The narrow A4 spike at ${\hat{E}}_{g}^{\mathrm{PBE}0}\approx 0$ corresponds to metal predictions that are pulled toward zero by the hurdle penalty. The figure is produced by the script which reuses the seeds and hyperparameters of Table 4; the violation count and $\delta_{\min}$ values printed on the panels coincide exactly with those in Table 4.

Figure 14 visualises the ablation contrast introduced numerically in Section 4.4 and Table 4: the unconstrained Neural ODE variant A3 places mass in the physically forbidden ${\hat{E}}_{g}^{\mathrm{PBE}0}<0$ region, while the full PC-NODE variant A4 does not, in agreement with Proposition 1.

Our headline result, a test MAE of 0.186 eV with a compact 34-dimensional descriptor set, sits close to the MXgap reference of 0.17 eV [11] and behind the 0.14 eV value reported by Tang et al. [10], a gap of at most 0.05 eV. The broader MXene–ML literature is itself moving rapidly: 2026 contributions extend ML-driven design to other functional regimes such as ML-guided microwave absorption optimisation of magnetic MXene composites [37], indicating that bandgap prediction is one node within a larger and active design ecosystem. The more informative comparison, however, does not pertain solely to the headline number, but also to what each pipeline gives up or preserves. Ontiveros et al. retain 33 elemental and 103 DOS-derived features (the latter including the PBE-level bandgap, band edges and averaged density of states), for a total of 136 inputs; Tang et al. train a deeper neural network inside a high-throughput screening pipeline. In contrast, PC-NODE uses only the 34 compact descriptors as model inputs and incorporates the PBE bandgap as the Δ learning baseline of Equation (2) rather than as an additional feature; this also avoids any post hoc monotonicity or positivity correction, because the first two of these properties are guaranteed by the architectural parameterisation, as stated in Proposition 1. A point on which our setting differs from the Zhang benchmark [12], where the classifier label IsGap is reported as the most important regression feature, is that our hurdle construction couples the two heads through the softplus parameterisation of Equation (2) rather than by feeding the label back as an input, which avoids the circularity of using the target as a covariate.

To position our framework more explicitly within the existing landscape of bandgap prediction methods, Table 7 compares PC-NODE with representative studies that span the major model families. The most directly comparable benchmark is Ontiveros et al. [11] on the same MXgap dataset and the same PBE0 target: their classifier–regressor pipeline (RF/GBT/MLP, with 33 elemental and 103 DOS-derived features—136 inputs in total—and a 60/20/20 split) attains a test MAE of 0.17 eV with 92% classification accuracy. Our PC-NODE ensemble reaches 0.186 eV with only 34 compact features as model inputs (no DOS or PBE-derived bandgap features) on a stratified 70/15/15 split, while additionally providing the architectural guarantees of Proposition 1. Tang et al. [10] use a deep CNN with feature fusion on a 23,857-structure aNANt+C2DB MXene set and report a bandgap MAE of 0.14 eV against HSE06 targets, so the discrepancy in dataset and functional precludes a strict shared-set comparison. The Zhang benchmark [12] reports MAE =0.022 eV and $R^{2}=0.985$ with a Gradient-Boosted Decision Trees (GBDT) model whose feature-importance analysis attributes 0.57 of the total importance to the binary IsGap flag itself; this confirms our earlier observation that feeding the classification label back as a regression input creates a target leakage path that PC-NODE deliberately avoids through the softplus hurdle parameterisation of Equation (2). Kernel methods are represented by Rajan et al. [8], whose Gaussian Process Regression on a curated subset of 70 semiconducting MXenes (drawn from a 23,870-structure functionalised MXene library) delivers a test RMSE of 0.14 eV against $G_{0}W_{0}$ targets; the small semiconductor sample size and the GW level rather than PBE0 target prevent a strict shared set comparison. Graph neural networks (GNNs) are represented by the Crystal Graph Convolutional Neural Network of Xie and Grossman [9], which reports a bandgap MAE of 0.388 eV on 16,458 Materials Project crystals across the periodic table; to our knowledge, no GNN benchmark dedicated to the open MXgap resource has been published, so an MXgap-specific GNN study is a natural follow-up. Finally, with respect to deep ensembles in the sense of Lakshminarayanan et al. [22], we note that the headline figures of this paper are themselves obtained from a ten-seed PyTorch ensemble; the per-seed standard deviation of 0.006 eV reported in Table 2 indicates that the ensemble is internally consistent.

The conformal results of Section 4.7 deserve a closer look. At the 0.90 nominal level, the empirical coverage is 0.905, a deviation of 0.5 percentage points that is smaller than the typical statistical fluctuation on a test set of a few hundred semiconductors. The 0.95 level slips to 0.926, which is expected given the finite calibration sample: the guarantee is valid in the infinite-calibration limit and becomes approximate in this regime. The mean interval width at 0.90 is roughly 1.05 eV, which is wide relative to the natural range of gaps in the dataset (median 0.67 eV among semiconductors) but is a faithful picture of the residuals the ensemble actually produces on unseen data. Importantly, these intervals are distribution-free: they do not rely on assumptions about the residual distribution, and they hold for any base estimator [18]. The complementary MC dropout spread reports the model-level, epistemic component of the uncertainty and is therefore expected to be narrower than the conformal interval, which captures aleatoric variation as well; as noted in Section 4.9, MC dropout is also known to underestimate predictive uncertainty on out-of-distribution inputs [34,35], so it should be read as an internal diagnostic rather than as a calibrated error bar.

The uncertainty-aware Pareto screening identifies Sc_4_N_3_O_2_ as the top candidate in the training library and then returns 74 lanthanum-based MXenes inside the photocatalytic window, led by La_2_CTe_2_, La_2_CCl_2_ and La_4_N_3_(NH)_2_. Our analysis restricts itself to the window criterion and the classifier confidence and does not attempt to validate these candidates with additional first-principles calculations. We also emphasise that the photocatalytic window is a *necessary* but not sufficient criterion: practical photocatalysis additionally requires appropriate band-edge alignment relative to the H^+^/H_2_ and O_2_/H_2_O redox levels, a supportive excitonic structure, and chemical stability of the terminal-group chemistry. The PC-NODE prediction on La_4_N_3_(NH)_2_, in particular, corresponds to a Δ correction of 1.813 eV starting from a zero PBE estimate, which sits in the upper tail of the training δ distribution and is accompanied by the largest MC dropout spread among the five top candidates in Table 6; we therefore flag this prediction as requiring independent validation.

Several limitations are worth making explicit. First, our training pool contains only eleven transition metals; the lanthanum-based screening provides an informative out-of-distribution test but is not fully adversarial, because lanthanum sits in Group III alongside Sc and Y, which are already in the training pool. A genuine adversarial test would require hybrid-functional calculations on compositions outside this family. Second, the compact descriptor set excludes the DOS by design; while we observed that adding DOS features or their PCA-compressed form did not improve the PC-NODE ensemble on this dataset, we cannot rule out that a larger training pool or a different regularisation strategy would benefit from DOS information. Third, the conformal guarantees rely on the exchangeability of calibration and test residuals, which holds under i.i.d. sampling but is not automatic for the lanthanum subset; weighted or nonexchangeable conformal variants [33] are a natural remedy that we leave to future work. Fourth, only two of the three physical properties we consider are enforced by architecture; the hurdle condition is penalised and therefore approximate, and we interpret the empirical metal prediction magnitude of 0.045 eV as acceptable rather than guaranteed. Fifth, the screening counts reported in Section 4.8 are obtained on the full 4356-structure pool, which overlaps with the training data, and should be read as a library re-scoring exercise; the lanthanum results of Section 4.9 provide the honest out-of-sample view.

To consolidate the discussion above into a single decision-oriented synthesis, we summarise the framework in the SWOT analysis of Table 8. The strengths group the architectural and statistical properties of PC-NODE that are verified within the present study; the weaknesses gather the items that the limitations paragraph has just enumerated; the opportunities translate the future work directions of the conclusions into actionable extensions; and the threats record the external factors that could constrain the practical impact of the framework, in the spirit of the methodological honesty that the reviewer is asking us to make explicit.

## 6. Conclusions

We have introduced PC-NODE, a physics-constrained neural ODE framework for the prediction and screening of MXene bandgaps on the MXgap dataset. The architecture couples a classifier head for the metal/semiconductor decision with a softplus-parameterised Δ learning regression head for the gap magnitude. Two physically motivated properties are obtained exactly by the parameterisation, as stated in Proposition 1: non-negativity of the predicted bandgap and monotonicity between the low-fidelity PBE estimate and the high-fidelity PBE0 prediction. A third property, the hurdle condition that metal predictions collapse towards zero, is enforced penally and verified empirically. This two-by-construction, one-approximately-enforced split is the formulation used consistently throughout the abstract, the methodology and the present conclusion. On a compact 34-dimensional descriptor set that excludes the DOS, a ten-seed ensemble reaches a test MAE of 0.186 eV and a coefficient of determination of 0.880, placing the model close to the reference MXgap benchmark with a substantially smaller feature footprint. Split-conformal calibration delivers prediction intervals whose empirical coverage matches the 0.90 nominal level within 0.5 percentage points, and the uncertainty-aware Pareto screening returns 74 lanthanum-based candidates inside the photocatalytic window [1.23,3.10] eV.

The mathematical content of the framework rests on three elements that are simple to state and verifiable in closed form. First, the architectural guarantees of Proposition 1 follow directly from the strict positivity of the softplus function and the clamping of the low-fidelity input, which together place every prediction in the admissible region $\left\{\left(\delta ,{\hat{E}}_{g}^{\mathrm{PBE}0}\right):\delta \geq 0,{\hat{E}}_{g}^{\mathrm{PBE}0}\geq 0\right\}$ of Figure 7. Second, the split-conformal quantile of Equation (4) furnishes finite-sample, distribution-free coverage at the nominal 1−α level, independent of the underlying neural surrogate. Third, the augmented Chebyshev scalarisation of Equation (7) ranks candidates by an explicit trade-off between the window distance and the classifier confidence, yielding a deterministic order on the candidate set. The empirical study of Section 4 supports each of these elements within the scope of the MXgap data.

Three directions appear natural for future work. The first is a more ambitious out-of-distribution programme, in which an independent hybrid functional dataset outside the present transition-metal family would be generated to stress-test the coverage of both the point predictions and the conformal intervals, together with the adoption of weighted or nonexchangeable conformal variants [33] that relax the exchangeability assumption. The second is the integration of symmetry-aware descriptors or graph-based encoders into the PC-NODE backbone, with the goal of making better use of the full-factorial design of the MXgap library. The third is experimental validation of the top-ranked lanthanum-based candidates through targeted synthesis and spectroscopic characterisation. Taken together, these directions would turn the present surrogate into a closed design loop for photocatalytic MXene discovery, with mathematical guarantees propagated end-to-end from the parameterisation to the ranked candidate list.

## Figures and Tables

**Table 1:** Fixed hyperparameters of the PC-NODE used throughout this study.

Component	Symbol	Value
Encoder hidden width	—	128
Latent dimension	*d*	64
Velocity field hidden width	—	128
ODE integration steps	*S*	3
Dropout rate	—	0.15
Optimiser	—	AdamW
Learning rate	—	$2\times 10^{-3}$
Weight decay	—	$3\times 10^{-4}$
Learning rate schedule	—	Cosine annealing
Batch size	—	128
Maximum epochs	—	300
Early-stopping patience	—	35 epochs
Gradient clipping norm	—	2.0
Regression weight	$\lambda_{\mathrm{reg}}$	1.5
Hurdle weight	$\lambda_{\mathrm{metal}}$	0.2
Monotonicity weight	$\lambda_{\mathrm{mono}}$	0.02
Ensemble size	—	10 seeds

**Table 2:** PC-NODE ensemble performance across descriptor sets (ten seeds). The per-seed column reports mean ± standard deviation across seeds; the ensemble column reports the metric of the averaged prediction. **Bold** values mark the best result within each column (lowest ensemble MAE and highest ensemble $R^{2}$).

Descriptor	Feat.	MAE/Seed (eV)	MAE Ens. (eV)	$R^{2}$ Ens.
Compact	34	0.206±0.006	**0.186**	**0.880**
Compact + DOS-PCA (12)	46	0.234±0.010	0.217	0.850
Full + DOS	134	0.254±0.007	0.233	0.832
Cross-descriptor mean	—	—	0.200	0.872

**Table 3:** PC-NODE ten-seed ensemble classifier performance on the shared test split. The confusion counts are on a test set of 654 structures.

Metric	Value
Accuracy	0.856
Macro $F_{1}$	0.810
Precision (semiconductor)	0.628
Recall (semiconductor)	0.831
$F_{1}$ (semiconductor)	0.715
ROC-AUC	0.925
PR-AUC	0.821
True negatives/false positives	442/70
False negatives/true positives	24/118

**Table 4:** Ablation study on the compact descriptor set (five seeds per variant; all variants share the optimiser, learning rate schedule, and maximum training epochs listed in Table 1). MAE is the mean ± standard deviation of the per-seed test MAE on the semiconductor subset. The ensemble MAE quoted in Table 2 (0.186 eV on ten seeds) is lower than the per-seed mean reported here (0.214±0.016 eV) because ensemble averaging reduces variance across seeds. Positivity violations count predictions with ${\hat{E}}_{g}^{\mathrm{PBE}0}<-10^{-6}$ on the test split; $\delta_{\min}$ is the minimum $\delta ={\hat{E}}_{g}^{\mathrm{PBE}0}-E_{g}^{\mathrm{PBE}}$ across seeds; metal $|\hat{y}|$ is the mean absolute predicted gap on metal structures (N/A for A1, which does not predict metals). Variants A2 and A4 satisfy the physics rails exactly; the unconstrained NODE variant A3 does not. **Bold** values mark either the best result within a column (lowest MAE, highest $R^{2}$) or the satisfaction of a physics rail (Pos. Viol. = 0, $\delta_{\min}\geq 0$).

Variant	MAE (eV)	$R^{2}$	Pos. Viol.	$\delta_{\min}$ (eV)	Metal $|\hat{y}|$ (eV)
A1 Plain MLP	0.248±0.008	0.822	0	N/A	N/A
A2 MLP + softplus hurdle	**0.203 ± 0.010**	**0.869**	**0**	**≥** **0**	0.242
A3 NODE (no physics)	0.218±0.011	0.849	37 (mean)	−0.588	0.389
A4 Full PC-NODE	0.214±0.016	0.845	**0**	**≥** **0**	0.214

**Table 5:** Split-conformal prediction intervals for the PC-NODE bandgap regressor.

α	Target 1−α	${\hat{q}}_{\alpha }$ (eV)	Empirical Coverage	Mean Width (eV)
0.20	0.80	0.410	0.847	0.784
0.10	0.90	0.572	0.905	1.051
0.05	0.95	0.699	0.926	1.237

**Table 6:** Top five lanthanum-based MXene candidates identified by PC-NODE, ranked by classifier confidence. Entries marked with † correspond to predictions whose MC dropout spread exceeds the cross-table mean and whose required Δ correction lies in the upper tail of the training distribution; these predictions require independent validation. The ✔ symbol in the “In Window” column indicates that the predicted PBE0 bandgap ${\hat{E}}_{g}^{\mathrm{PBE}0}$ falls within the photocatalytic water-splitting window [1.23,3.10] eV.

Structure	$E_{g}^{\mathrm{PBE}}$ (eV)	${\hat{E}}_{g}^{\mathrm{PBE}0}$ (eV)	MC σ (eV)	$\hat{p}$ (Semi)	In Window
La_2_C_1_Te_2_	1.393	2.924	0.118	>0.999	✔
La_2_C_1_Cl_2_	0.457	1.986	0.073	>0.999	✔
La_4_N_3_(NH)_2_^†^	0.000	1.813	0.185	>0.999	✔
La_2_C_1_Se_2_	1.309	3.013	0.119	>0.999	✔
La_3_N_2_(NH)_2_	0.126	2.066	0.122	>0.999	✔

**Table 7:** Positioning of PC-NODE relative to representative bandgap-prediction methods across the major model families. All numerical values are taken from the cited references. The metric column reports MAE unless specified otherwise; differences in dataset, target functional and split protocol are noted in the third column and discussed in the surrounding text.

Method	Family	Dataset (Size; Target)	Features	Reported Metric
PC-NODE (this work)	Neural ODE + Δ learning + 10-seed ensemble	MXgap (4356; PBE0)	34, no DOS	MAE 0.186 eV; $R^{2}$ = 0.880; acc. 0.856
Ontiveros et al. [11]	Classifier–regressor (RF/GBT/SV/MLP)	MXgap (4356; PBE0)	33 elemental + 103 DOS-derived (136 total)	MAE 0.17 eV; acc. 0.92
Tang et al. [10]	Deep CNN with feature fusion	aNANt + C2DB (23,857; HSE06)	layered-image + atomic features	MAE 0.14 eV
Zhang [12]	GBDT/LightGBM/SVR	MXene database (4488; PBE0)	12 incl. IsGap as input feature	MAE 0.022 eV; $R^{2}$ = 0.985
Rajan et al. [8]	Gaussian Process Regression (kernel)	70 semicond. MXenes from 23,870 lib.; $G_{0}W_{0}$	elemental + radii + bond lengths	RMSE 0.14 eV
Xie & Grossman [9]	Crystal Graph CNN (GNN)	Materials Project (16,458; DFT)	atom/bond graph	MAE 0.388 eV

**Table 8:** SWOT analysis of the PC-NODE framework. Internal factors (strengths and weaknesses) describe properties of the present study; external factors (opportunities and threats) describe the wider context in which the framework will be deployed.

Strengths (Internal, Positive)	Weaknesses (Internal, Negative)
Two of the three physics constraints are exact by architecture (Proposition 1: non-negativity and PBE/PBE0 monotonicity).	The hurdle condition is enforced as a soft penalty rather than by construction; the empirical metal residual is small (0.045 eV) but not architecturally zero.
Distribution-free split-conformal intervals; empirical coverage 0.905 at the 0.90 nominal level (Section 4.7).	Mean conformal interval width of ≈1.05 eV at the 0.90 level is wide relative to the median semiconductor gap (0.67 eV) on the test set.
Compact 34-dimensional descriptor with no DOS or PBE-bandgap input feature; the PBE estimate enters only as the Δ learning baseline of Equation (2), which avoids any target-leakage path.	Training pool covers only eleven transition metals; the lanthanum out-of-distribution test is informative but not fully adversarial.
Continuous-depth Neural ODE backbone with multi-fidelity Δ learning; ten-seed ensemble with per-seed MAE standard deviation of 0.006 eV (Table 2).	Screening counts are obtained on the full 4356-structure MXgap pool that overlaps with training and should be read as library re-scoring rather than out-of-sample discovery.
Open-source pipeline trained on the public MXgap resource, with reproducible splits and hyperparameters.	DOS information is excluded by design; subtle electronic features that may matter for some MXene families are not captured.
Adoption of weighted or nonexchangeable conformal variants [33] to extend coverage guarantees beyond strict exchangeability and into out-of-distribution regimes.	Distribution shift towards compositions far outside the MXgap chemical envelope could degrade the empirical conformal coverage in regimes where exchangeability is no longer plausible.
Integration of symmetry-aware descriptors or graph-based encoders into the PC-NODE backbone, with the goal of exploiting the full-factorial design of the MXgap library.	Differences in target functional across studies (PBE0 here, HSE06 in Tang et al., $G_{0}W_{0}$ in Rajan et al.) limit strict shared-set comparison and complicate cross-study benchmarking.
Generation of independent hybrid functional benchmarks outside the present transition-metal family, providing a genuinely adversarial out-of-distribution test bed.	Computational cost of hybrid-DFT validation on the top-ranked lanthanum candidates may delay independent confirmation of the surrogate predictions.
Experimental validation of the top-ranked lanthanum-based candidates (La_2_CTe_2_, La_2_CCl_2_, La_4_N_3_(NH)_2_) via targeted synthesis and spectroscopic characterisation.	Larger GNN-based models trained on broader 2D-materials repositories may eventually outperform compact descriptor approaches on cross-family generalisation, even at higher inference cost.
Extension of the framework to other 2D and functional-materials families where multi-fidelity data and physics constraints can be formulated in the same mathematical form.	Limited semiconductor sample size in genuinely out-of-distribution regimes inflates the conformal interval and may render some predictions practically uninformative without further data acquisition.

## Data Availability

The MXgap dataset [11] is publicly available via the corresponding Zenodo record (DOI: https://doi.org/10.5281/zenodo.14858915, accessed on 24 May 2026); to avoid duplicating the original distribution, we do not re-host the raw data. The machine learning code, the data-processing scripts, and the train/validation/test split indices that support this study are openly released at https://github.com/drferhatu/PC-NODE-MXene-Paper (accessed on 24 May 2026), with a permanent archive on Zenodo (DOI: https://doi.org/10.5281/zenodo.20398091, accessed on 24 May 2026).
